# Low-Carbohydrate Diet and Human Health

**DOI:** 10.3390/nu15082004

**Published:** 2023-04-21

**Authors:** Sousana K. Papadopoulou, Pantelis T. Nikolaidis

**Affiliations:** 1 Department of Nutritional Sciences and Dietetics, School of Health Sciences, International Hellenic University, 57001 Thessaloniki, Greece; 2 School of Health and Caring Sciences, University of West Attica, 12243 Athens, Greece

Low-carbohydrate diets were initially recommended as a therapeutic dietary scheme for epilepsy, while increasing evidence suggests their potential application in the management of several other pathologies, such as diabetes, neoplasms, gastrointestinal and lung diseases, diseases of the cardiovascular system, as well as obesity. For the present editorial, four manuscripts were gathered, including two systematic reviews and two research articles. Novel knowledge is presented regarding the adoption of low-carbohydrate diets in older adults, people with chronic diseases, children, and those living in a low socio-economic environment, with special focus on patients’ quality of life, disease prevention, and nutritional coverage. Further studies are needed to identify the patients’ and general population subgroups that could benefit from a low-carbohydrate diet in a personalized approach.

Low-carbohydrate diets were firstly recommended as a therapeutic dietary scheme for epilepsy [1]. In these diets, carbohydrates represent only 5–10% of total energy (10–50 g) [2], while fat and protein quantities vary [2]. Moreover, several versions of low-carbohydrate diets have been introduced, such as the Atkins diet, the modified Atkins diet, the ketogenic diet, the modified ketogenic diet, the very low calorie ketogenic diet, and the ketogenic Mediterranean diet, which allow a higher consumption of carbohydrates (<30–50 g per day) compared to other diets [2].

Low-carbohydrate diets are related to the genesis of ketone bodies due to fatty acid oxidation and the upregulation of ketogenic enzymes [3], appetite suppression [4], improved postprandial glucose metabolism [5], and a reduction in insulin-like growth factor 1, which is implicated in cancer [6]. In this context, increasing evidence suggests their potential application in several pathologies other than epilepsy, such as diabetes, neoplasms, gastrointestinal and lung diseases [5], diseases of the cardiovascular system [7], as well as obesity [8,9]. The actual carbohydrate content of the diet may affect the potential observed benefits. For example, moderately low carbohydrate or low-carbohydrate diets may be useful for weight reduction, while a very low carbohydrate diet may not be ideal for patients with diabetes [10], and it may adversely affect the lipid profiles in this subgroup [11]. However, in a recent randomized clinical trial, both diets had comparable effects in obese subjects with metabolic disorders in the short term (2 months) [12]. Some studies have also addressed the potential effects of low-carbohydrate diets on quality of life [13], which may be worsened in chronic diseases affecting physical status, psychology, and chronic stress levels [14,15].

Indeed, in the present Special Issue, a systematic review of Abboud et al. [13] considered the available evidence of nine randomized controlled trials regarding the effect of the ketogenic diet on the quality of life in subjects with chronic diseases. A satisfying dietary compliance was reported, but only three studies provided such information [13]. Although evidence on quality of life was inconclusive, some studies showed promising results with low side-effects following a ketogenic diet, implying that there is a need for future studies in this field [13].

The potential health effects of a moderate carbohydrate diet were systematically reviewed by Papadopoulou et al. [16], who focused on the relationship between the Mediterranean diet and sarcopenia in older adults (>65 years old). This aspect is important since, up to now, most studies evaluating the role of diets in sarcopenia have focused on specific nutrients [17,18]. More particularly, no randomized controlled trials were identified in relation to this topic [16]. The cross-sectional (n = 4) and prospective (n = 6) studies available revealed that the Mediterranean diet may positively affect muscle mass and function [16]. However, the data were not clear with respect to muscle strength, and no positive effect of the Mediterranean diet on sarcopenia was found [16]. It is noted that the systematic review of Papadopoulou et al. excluded patients with comorbidities [16]. This is important to consider in light of the evidence that dietary patterns can affect body composition [19].

Several concerns have been raised regarding the nutrient content of low-carbohydrate diets, while initial studies in epileptic children draw connections to osteopenia [20]. High-fat food choices of possibly lower nutritional value are usually preferred in low-carbohydrate diets [21]. This issue may be more important in children and adolescents who have rapid physical growth and special nutritional needs [22]. In this Special Issue, Zinn et al. designed menus that were analyzed with nutritional software, which provided ≤80 g of carbohydrates and 15–25% of energy from proteins [22]. They illustrated that appropriately designed meal plans for children can provide adequate amounts of energy, protein, and micronutrients [23]. Indeed, nutritional algorithms [24], as well as carefully designed menus, can help achieve nutritional goals according to guidelines, even in disease states [25]. Similarly, a recent review of intervention studies underlined that the adoption of the ketogenic diet related to increases in circulating vitamin D [26]. However, saturated fat intakes may be problematic in some cases, mainly due to the inclusion of high fat diary; thus, proper tailored-made personal dietary plans are needed [23].

Last but not least, several factors may contribute to the initiation of this pattern [27] and long-term adherence [28]. For example, when beginning a low-carbohydrate diet, several symptoms may be present, such as nausea and vomiting, headaches, and hypoglycemia, which may reduce adherence [3,6]. A qualitative study by Pujol-Busquets et al. published in this Special Issue presents the facilitators and challenges in following a low-carbohydrate approach in a low socio-economic status context [29]. Indeed, women participating in this study were receptive to a low-carbohydrate intervention, but some of them expressed concerns and dietary change was considered challenging in their socio-economic setting [29].

In conclusion, this Special Issue provides novel knowledge regarding the adoption of low-carbohydrate diets among older adults, people with chronic diseases, children, and those living in a low socio-economic environment, with special focus on patients’ quality of life, disease prevention, and nutritional coverage. Further studies are needed to identify the patients and population subgroups that could benefit from a low-carbohydrate diet in a personalized approach. Long-term effects of such diets should also be investigated [30].
